# Innovative Noninvasive HPV Screening Using a Feminine Pad: A Pilot Study for Enhanced Cervical Cancer Detection

**DOI:** 10.1155/ogi/9344596

**Published:** 2025-04-28

**Authors:** Rodrigo Aldariz-Amaya, Miriam Rodríguez-Esquivel, Alfonso Ruiz-Romero, Fernanda Anthor, Emmanuel Mares, Angélica Flores-Martínez, Edgar Alejandro Gómez-Villa, Marco Antonio Fuentes-Garcia, Teresa Apresa-García, Ricardo López-Romero, Emmanuel Salcedo, María de Jesús Nambo-Lucio, Mauricio Salcedo

**Affiliations:** ^1^Unidad de Investigación en Biomedicina y Oncología Genómica, Hospital de Gineco-Pediatría 3A, OOAD CDMX Norte, Instituto Mexicano del Seguro Social (IMSS), Mexico City, Mexico; ^2^Unidad de Posgrado, Departamento de Ciencias Biológicas, UNAM, Ciudad Universitaria, Edificio A, Piso 1, Coyoacán, C.P. 04510, Mexico City, Mexico; ^3^Department of Physiology and Biophysics, The University of Chicago, Chicago, Illinois, USA; ^4^División de Ginecología, Hospital de la Mujer, Secretaria de Salud, Mexico City, Mexico; ^5^Unidad de Investigación Médica en Enfermedades Oncológicas, Hospital de Oncología, Centro Médico Nacional Siglo XXI-IMSS, Mexico City, Mexico; ^6^Room 2, Hospital Angeles Clinica Londres, Colonia Roma, Mexico City, Mexico

**Keywords:** detection, device, HPV, noninvasive, obstacles

## Abstract

**Background:** Human papillomavirus (HPV) is a major contributor to cervical cancer (CC), with Papanicolaou (Pap) smears and HPV testing serving as primary screening tools in developed countries. The effectiveness of these methods can vary based on a country's epidemiological and socioeconomic context. This study introduces an innovative, noninvasive method employing surgical gauze worn as a feminine pad for HPV detection, with the aim of simplifying and improving screening processes.

**Materials and Methods:** A total of 184 participants, including individuals classified as healthy, with cervical precursor lesions, or as with confirmed CC, were enrolled. All participants underwent cytological and colposcopic evaluations, with biopsies taken in cases of abnormal results. Each participant wore the device for 8 h, after which DNA was extracted from the soiled devices and analyzed via PCR for mitochondrial and HPV–DNA. Sensitivity and specificity were calculated to assess the effectiveness of HPV detection. Sensitivity and specificity values for HPV detection were obtained. Analysis of diagnostic tests was performed by OpenEpi software.

**Results:** The device was well-received, with high compliance among participants. PCR analysis revealed that 17.7% of healthy, 72.1% of precursor-lesion cases, and 97.1% of CC cases tested positive for HPV. The calculated sensitivity and specificity for detecting high-grade lesions (CIN2+ or CIN2/3 and CC) were 94.81% and 51.28%, respectively. For CC detection, sensitivity was 97.14% with a specificity of 54.39%.

**Conclusions:** The use of this noninvasive device demonstrated a significant correlation with clinical outcomes, supporting its potential as an effective and accessible tool for HPV screening. This method offers a promising alternative to conventional screening techniques, particularly in settings where traditional methods face logistical and socioeconomic challenges.

## 1. Introduction

Cervical cancer (CC) remains a preventable disease marred by significant global disparities in its prevention. By 2030, new cases are projected to reach 700,000 annually, predominantly in the world's poorest countries, where they often result in death [1]. This trend can largely be attributed to inadequate awareness of the disease, substantial socioeconomic obstacles, and the absence of effective health coverage or well-organized screening initiatives [1, 2]. Consequently, there is a critical need to reevaluate gynecological procedures and address the barriers impacting women's understanding of CC and their perceptions of Papanicolaou (Pap) smear screening [3].

Several studies now show a widespread perception of the Pap smear as an uncomfortable and embarrassing event during the procedure, becoming particularly painful when the speculum is not handled properly and is of the wrong size. For the women, this is a test that is “not seen but is felt,” generating feelings of vulnerability (for review, see [4]). Sometimes women mention that the screening tests are not affordable, and moreover, they always complained of the long waiting period [3]. These studies suggest that is necessary to design novel devices for the future implementation and acceptability in women for CC screening. This will largely depend on the woman's comfort during the test and the timely delivery of results [3].

Epidemiological studies indicate that most patients with CC are or were infected with human papillomavirus (HPV) [5]. Additionally, the precursor cervical intraepithelial lesions or CIN2+ or high-grade squamous intraepithelial lesions (SILs), mainly attributable to persistent HPV infection, can become an early pathognomonic sign of CC [6–8]. Thus, the lack of high-quality screening programs could also explain the high frequency of HPV infection [9–11], exacerbating the importance of HPV screening methods as a crucial step in finding HPV-cervical lesions at an early phase.

The Pap cervical smear test is an examination applied to detect cellular abnormalities [12, 13], but it is not an ideal gold standard due to its subjectivity and to its relatively low accuracy and low sensitivity. Although it is relatively inexpensive and simple, it entertains at least the caveat of being invasive and uncomfortable. Due to the low perception of the Pap smear, the majority of women would prefer less invasive screening benefits [14]. For this, some crucial steps in the sampling process should be considered [15, 16].

At present, different options being sought for the collection of cervicovaginal samples to reduce costs and barriers are being reported (for review, see Ref. [17–19]). But their cost, acceptability, and scalability as part of population-level screening programs need to be considered [20, 21].

Interestingly, a small number of studies have reported “strictly noninvasive” procedures by utilizing different biofluids such as menstrual blood, vaginal discharges, or urine as alternatives in HPV detection for CC screening [22–25]. These reports suggest that the self-collection of different biofluids (such as blood or urine), together with the HPV test, could be a key strategy for the successful upscaling of cervical screening programs.

We previously reported the use of commercial surgical gauze employed as a feminine pad (or medical device) for a noninvasive detection of the volatile organic compounds (VOCs) [26], as well as CC-related compounds, and also as an appropriate source and collector system for DNA extraction/analysis. Following this innovative strategy, in the present work, we set out to demonstrate the feasible detection of HPV sequences from the devices.

## 2. Materials and Methods

### 2.1. Ethical Statement

The Scientific and Ethics Committees of the Mexican Institute of Social Security (IMSS) (Comité Nacional de Investigación Científica del Instituto Mexicano del Seguro Social and deriving from Hospital de la Mujer, SS, Mexico City, HM-INV-007-2019) approved this study, and all samples were taken from study participants after they signed an informed consent document. Interestingly, all participants accepted to wear the device among all the women we approached, none refused to participate.

### 2.2. Population Study

The present work was a pilot, cross-sectional, observational, and analytical study following the Strengthening and the Reporting of Observational Studies in Epidemiology (STROBE) statement [27]. Healthy and women with SIL were selected from a cohort of patients from the Dysplasia Clinic of the Hospital de la Mujer (HM), SS, Mexico City (prior to the COVID-19 pandemic) during routine gynecological inspection and Pap testing. Patients with CC were selected from the Gynecology Service, Hospital de Oncología, Centro Médico Nacional SXXI-IMSS (HO-CMNSXXI, IMSS) in Mexico City during the 2017–2019 period who sought proper therapy treatment.

**Figure 1:**
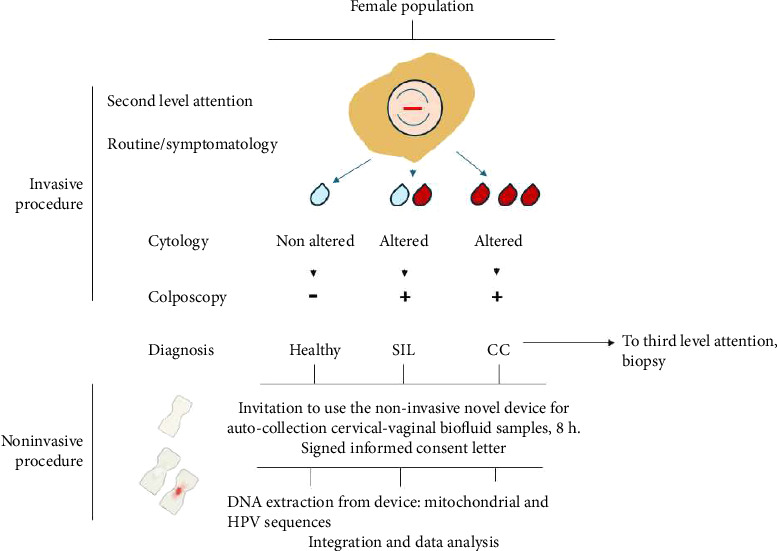
Fluxogram of the implemented device strategy. All patients with abnormal cytology were referred to second level of attention for colposcopy examination; these are two invasive procedures. After colposcopy examination, the patients were classified as healthy (without any cervical lesions) or sick (with SIL or CC). For this phase of the strategy, the patients were invited to wear the device and they signed an informed consent letter. Finally, the DNA purified from the device was subjected for mitochondrial or HPV detection. A uterine cervix creative image is observed. The clear drops show healthy Pap smear without any symptomatology. The dark drops are indicating altered Pap smear with symptomatology. Also, a creative image of the clean or soiled device is observed.

Figure 1 presents the strategy followed in the present study. All selected women were gynecologically and cytologically evaluated by Pap smear, a strict invasive procedure. For the Pap smear, the endo- and exocervical cells were scraped with a cytobrush, smeared onto a glass slide, fixed, and stained. In the case of abnormal cytology, patients were referred for a colposcopy-5% acetic-acid assessment, a colposcopy-guided biopsy for histologic examination. “Healthy”-status patients were also colposcopically evaluated for the present study. Their cytology was classified according to the Bethesda system [28]—by a cytologist, while for a biopsy sample classified as precursor or invasive, this was classified by a pathologist. All Pap smears and biopsies were analyzed immediately after collection. This study was conducted only after obtaining and reviewing the results from cytology, colposcopy, and histological examinations. Clinical and sociodemographic data were recorded during each visit.  Table 1 displays the clinical data for patients with SIL and CC.

The study included 184 consecutive participants aged over 18 years: 107 were classified as normal or low-grade intraepithelial neoplasia (CIN1-), seven had high-grade lesions (CIN2/3), and 70 were diagnosed with CC. Each participant used the device, which was subsequently analyzed. Due to the closure of laboratories during the COVID-19 pandemic, these devices were stored at −70°C for periods ranging from 4 months to 1 year.

### 2.3. Using the Device

**Figure 2:**
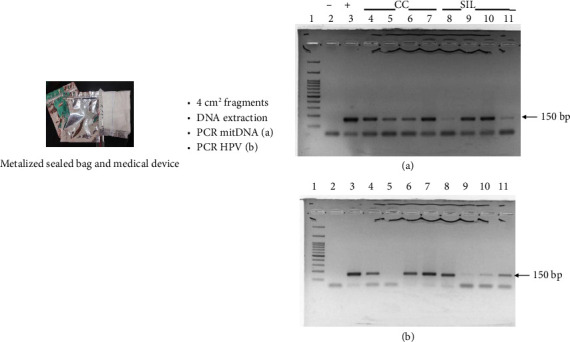
The cervical device, mitochondrial DNA, and HPV–DNA sequences detected from the soiled device. It shows a picture of the device and the metalized sealed bag containers employed. (a) Upper right gel. Purified DNA from a small fragment of the devices was subjected to PCR for mitochondrial DNA. Lanes: (1) molecular weight marker 100 bp ladder; (2) no DNA, as negative control; (3) HeLa DNA as positive control; (4–7) CC DNA from devices; and (8–11) SIL DNA from devices. All samples are showing mitDNA sequence; lanes 7 and 11 with slight perceptive band. The low intensity of the bands could suggest probably differences in the time of use of the device. The arrow shows the mitDNA band (150 bp band). Then, DNA samples were subjected for HPV/DNA detection. (b) Lower right gel. Lanes: (1) molecular weight marker 100 bp ladder; (2) no DNA, as negative control; (3) HeLa DNA as positive control; (4–7) CC DNA from devices; (8–11) SIL DNA from devices. Heterogeneity HPV band is observed. Even when 8 and 11 lanes showed slightly perceptive mitDNA band, a clear HPV band is noted. A CC sample HPV negative (Lane 5), while a slightly perceptive band was observed for some samples (lanes 9 and 10). The extremely low intensity of HPV bands could represent a very low viral load. The arrow shows the HPV–DNA band (150 bp). The bottom bands indicate excess of oligonucleotides or low DNA concentration; in all cases, the PCR did work properly.

Participants were instructed in the use of the “medical device” (Figure 2) and were asked to wear it as a sanitary pad overnight at home (for at least 8 h). The following morning, the device was deposited in a sealed individual packaging metalized bag in which the used device was returned. The collection device was provided by the researchers as part of the study.

### 2.4. DNA Eluted and Purified From the Device

Genomic DNA was extracted and purified employing the following steps: first, the area of maximal biofluid concentration was selected and cut from the device (4 cm^2^ from each). This piece was transferred into a microtube containing 1 mL of PBS buffer, which was during 10 min at room temperature, and then, a fragment of the device was removed. Once the microtubes were cleared of debris, 300 *μ*L of nuclear lysis buffer (10 mM Tris-Cl pH8, 1 mM EDTA pH8, 0.1% (w/v) SDS) and 10 μL of proteinase K solution (10 μg/mL) (ThermoFisher Scientific Co., NY, USA) were added and incubated overnight at 55°C. Then, 300 μL of 5 M ammonium acetate was added. Subsequently, the DNA was precipitated with isopropanol, washed with 70% ethanol, and resuspended with DNAase-free water. DNA quantity and purity were measured with a Nanodrop ND-1000 spectrophotometer (ThermoFisher Scientific, Co.).

### 2.5. PCR for Human Mitochondrial DNA (mitDNA) Amplicon and HPV Detection

Briefly, to demonstrate whether the DNA extract presented the quality necessary for an amplification reaction, DNA purified from the device was first subjected to PCR utilizing D-loop mitochondrial primers (internal control) [29]. All utilized devices comprised mitDNA exhibiting a 150-bp (base pair) band. Another aliquot of DNA was challenged for HPV–DNA detection. Each 100 ng DNA in a final volume of 20 μL was subjected to HPV detection by PCR employing GP5+/GP6+ primers [30]. These oligonucleotides amplify a 150-bp fragment inside a highly conserved region of the HPV/L1 viral gene. This pair of oligonucleotides has been widely used because the oligonucleotides possess the potential to detect low- and high-risk HPV types. Its amplicon length permits a very good PCR reaction result, avoiding any rearrangement of extreme degradation for the viral DNA. Finally, the PCR products were visualized via electrophoresis with 1.5% agarose bromide-stained gel.

### 2.6. Data Analysis

All collected data were encoded in a data source in the Microsoft Excel. The data were analyzed using descriptive statistics to define sociodemographic and clinicopathological features. The statistical parameters were obtained by using the OpenEpi software platform [31] and also with the MedCalc platform (https://www.mdcalc.com/#Popular); analyses of the diagnostic tests were performed with a 95% confidence interval (95% CI).

## 3. Results

### 3.1. Initial Impressions and Acceptability of the Device

The recruited women were asked to wear the device for at least 8 h daily. Despite some initial reservations, the device was well-received and considered a preferable alternative to traditional methods involving the vaginal speculum. The participants valued the noninvasive nature of the device, which aligns with the sensitive nature of gynecological health products. Notably, no challenges or discomforts were reported by any of the participants during the use of the device, underscoring its potential as a novel method for cervical health screening.

### 3.2. Cytological, Colposcopy, and Histological Findings

Findings from cytology, colposcopy, histology, and HPV detection in cervical lesions are detailed in Table 2. Each participant underwent a Pap smear and colposcopy; those with suspected SIL or CC also received a biopsy for histological analysis. Initial cytology results identified 48 samples as negative for intraepithelial lesion or malignancy (NILM), eight as koilocytosis, 12 as atypical squamous cells of undetermined significance (ASC-US), and 32 as low-grade SILs (LSILs). Further colposcopy and biopsy assessments revealed 53 negative outcomes, 10 condylomas, and 44 cases of CIN1. The ASC-US results, often influenced by technical variability or artifacts, were mostly reclassified upon colposcopy as CIN1-, with some reclassified as normal and two of these as CIN2/3. These findings affirmed strong correlation and precise classification, especially for cases progressing beyond CIN1-. The study grouped the 184 participants into three categories for analysis: 107 as normal or low-grade (CIN1-), seven as high-grade lesions (CIN2/3), and 70 were diagnosed with CC, aligning with the stratified risk approach in CC screening programs.

### 3.3. mitDNA and HPV–DNA Detection From the Devices

Each device was visually inspected to confirm proper use, and this serving as an initial quality control measure. DNA was subsequently extracted from the worn devices and analyzed using PCR to detect both mitDNA and HPV–DNA. Due to the COVID-19 pandemic, many laboratories were closed, requiring the samples to be stored at −70°C for durations ranging from the following day up to 1 year. Despite these challenging conditions, we successfully detected HPV sequences from the devices, yielding reliable results.

Genomic DNA (300 ng) was extracted and purified from small pieces of the devices used. A mean of 1.7 purity values was obtained, and the DNA yield was roughly 20 ng/*μ*L; the PCR tests were performed with 100 ng of genomic DNA in a 20-μL final volume. First, the DNA obtained was tested for mitDNA detection as the process' internal (biological) control. The outcomes revealed that all devices consistently displayed mitDNA sequences by PCR (Figure 2(a)). The stained gel revealed a representative result where all samples demonstrated a 150-bp band. Occasionally (as in lanes 8 and 11), weak bands are observed, suggesting a low concentration of mitDNA, probably due to a lesser time of pad exposure.

Following the strategy, the DNA was tested for HPV. Overall, there were 120/184 (65%) HPV-positive samples. Specifically, 8/53 (13.3%) of normal (healthy) patients were HPV-positive, 44/61 (72.1%) for precursors, and 68/70 (97.1%) for CC devices. Figure 2(b) is a representative result for a HPV test. We observed an HPV-negative CC sample (Lane 5) and also noted that there were some samples with a weak band in comparison with the other samples. This lack of intensity could be explained by an extremely low viral load. The HPV data showed good correspondence with the colposcopy and histology classification. It is noteworthy that only two CC that were histologically confirmed tested negative for HPV sequences.

### 3.4. Sensitivity and Specificity Analysis

Following the analysis and to know the sensitivity and specificity values, the HPV data were compared with cytology/colposcopy data for SIL or CC cases. CC samples revealed 89.4% (95% CI: 80.3–95.3) and 95.7% (95% CI: 85.46–99.2), respectively, with two false positives and eight false negatives. There is no doubt that false positives indicate the misclassification or misinterpretation of test results, probably due to an extremely low viral load or to a brief use of the device. To precise the data, the sensitivity and specificity values for CIN2+ and another for cancer were calculated (Table 3). For CIN2+, the sensitivity (94.8%) and specificity (51.28%) were obtained, while for CC (97.14%) and (54.3%), respectively. The accuracy of HPV detection for NIC2+ was 68.56% (95% CI: [61.52–75.02]) or 70.65% (95% CI: [63.50–77.12]) for CC samples.

## 4. Discussion

In the present work, we show that wearing surgical gauze as a feminine pad for at least 8 h can detect HPV sequences representing a strictly alternative noninvasive procedure regarding standard classical cytology.

The goal of CC screening programs is to identify high-grade precancerous lesions or CIN2+ in vulnerable population who can be treated before they progress to CC; however, the high prevalence and mortality of CC for the poorest countries suggest several deficiencies in screening programs [1]. Furthermore, it is well recognized that the lack of the routine gynecological examination is strongly associated with several obstacles, including social, cultural, psychological, and religious, in the under- or never-screened female population [2–4]. The role of HPV in CC has been widely demonstrated, and several molecular tests have been developed for the detection of this virus [32]. Epidemiological data indicate that HPV genotype distribution and prevalence vary depending on the geographic region [33]; this information is relevant for the specificity of the HPV test. For example, it was documented that the specificity of the HPV test was lower in South Africa than in Italy (81.9% vs. 94.2%) [34].

In Mexico, regional variations in HPV prevalence pose significant public health challenges [35]. Currently, CC screening primarily relies on Pap smears, which exhibit a sensitivity of 53% and a specificity of 85%; these figures suggest that the effectiveness of Pap smears in detecting CC may be adequate [36]. Notably, cost-effectiveness analyses highlight that HPV testing could serve as a viable screening alternative within large healthcare delivery organizations in Mexico [37], underscoring the need for innovative approaches in HPV detection.

A wide array of HPV testing methods [32], including several invasive sampling techniques and the use of biofluids such as urine and menstrual blood, has been documented [15–17, 32]. These developments indicate promising avenues for improving access to HPV screening, particularly for populations difficult to reach. Importantly, studies have shown that self-collected samples using various types of brushes for HPV testing align well in terms of specificity (over 90%) with those collected by medical professionals using PCR-based tests [20, 21]. This consistency supports the potential for self-collection methods to play a crucial role in enhancing screening accessibility and compliance tailored to meet specific national or regional health objectives [22].

Noninvasive methods such as menstrual blood collection have demonstrated sensitivities between 82.8% and 97.7% and specificities ranging from 50% to 98% in detecting SIL or HPV infection [23]. Urine collection methods demonstrate similar clinical accuracy with sensitivity ranging from 54% to 99% and a specificity between 67% −99% [25]. These findings suggest that HPV testing employing urine or menstrual blood may be viable screening alternatives for detecting cervical lesions. Notably, a single study using a sanitary pad reported a sensitivity of 76.9% and a specificity of 97.8% [24], aligning with our findings and supporting the potential of our device as a comparable, noninferior method for HPV detection. As expected, the present sensitivity of HPV detection data is different from those of Africa or Italy [34]. In this context, the present initiative of the device worn as a feminine sanitary pad is strengthened as an alternative for HPV detection. This could enable the elimination or reduction of obstacles that avoid many women from presenting for CC screening, in addition to obstacles also for health, technical, and/or related personnel.

Our device, designed to collect a mix of female anogenital biofluids—including urine, vaginal discharge, and blood (menstrual or not)—has proven effective in identifying HPV–DNA, with efficiency rates exceeding 90% [24, 26, 38]. This suggests that the device could serve as a novel tool for collecting cervicovaginal biofluids, thus enhancing CC screening and HPV detection capabilities. The robust correlation between clinical evaluations and HPV detection outcomes from our device indicates its utility in regions with high incidences of CIN2+, potentially overcoming traditional barriers to screening related to healthcare infrastructure and personnel constraints.

By capitalizing on the device's ability to collect a comprehensive range of biofluids, we can improve the accuracy and reach of HPV screening, making it a valuable tool in global health contexts where CC remains a prevalent threat.

Our device is designed as a strictly noninvasive, auto-collection tool that efficiently gathers optimal biofluids for HPV detection. It is comfortably worn for at least 8 h, acting as an excellent source of genital-tissue DNA and biomolecular markers associated with carcinogenesis, such as VOCs and HPV sequences. The device effectively concentrates even samples with extremely low viral loads, ensuring accuracy, reliability, and environmental friendliness. Additionally, its commercial cost is less than one U.S. dollar, rendering it a cost-effective option for widespread use. Regarding storage, the device's capacity to preserve genomic DNA under ultra-freezer conditions for up to 1 year enhances its suitability for PCR detection. DNA stability within the microenvironment of the device supports the hypothesis that it can detect DNA sequences shorter than 500 base pairs. The use of GP5+/GP6+ primers, recognized for their precision in HPV detection and included in the latest list of United States Federal Drug Association (FDA)–approved assays for primary CC screening [39], corroborates the device's efficacy.

Given that this study was conducted in a hospital setting, there is the need to expand research into population-based studies, particularly among previously unscreened populations, to further validate and implement this screening strategy. The sensitivity and specificity data, as well as the optimal negative and positive predicted values obtained in the current work, are intriguing with respect to further investigation. The data suggest that this is a reasonably reliable test for detecting an HPV true positive, particularly in NIC2+ cases, and recommended additional clinical procedures, such as colposcopy, to confirm the result. The outcome of the clinical data regarding the HPV detection (accuracy and effectiveness) observed in the present work reveals a well-established, significant association indicating the likelihood of practical use.

The small number of samples studied demonstrates the need for more investigation; this small number is a weakness of our work. However, we are convinced that the present device is promising and that it will be the preamble of a revolutionary alternative strategy for the benefit of the female population, both rural and urban. We think that the lack of comparison with any other self-sampling device [40] does not comprise weakness. At present, this comprises an ongoing study to improve our findings and to make them more robust.

Furthermore, we do not rule out the possibility of wearing the device for posttreatment follow-up, and the surveillance of patients with CC. Finally, on wearing the device, the endo- and exocervical tissues remain intact, making them available for future cytology smears and colposcopy investigations. The healthcare system and its policymakers must investigate innovative screening tests to improve CC screening.

## 5. Conclusions

In summary, this study serves as a proof-of-concept for a noninvasive, self-collecting method that enhances HPV detection. The device, which can be worn on any day of the menstrual cycle, continuously collects epithelial cells and biofluids through natural gravity. This innovative approach not only simplifies the collection process but also expands the potential for broader research applications in the field of cervical health. This method could significantly contribute to the early detection of HPV and potentially impact CC screening strategies globally.

## Figures and Tables

**Table 1:** Clinical data of women with squamous intraepithelial lesions and cervical cancer using the device.

	*n*	*p*	HPV +/− (*n*)
Age (y)			
< 30	36		23/13
> 30	38	0.8162	24/14
Body mass index			
< 25	30		22/8
> 25	44	0.1036	25/19
Menarche (y)			
< 12	13		9/4
> 12	61	**< 0.0001**	36/25
Beginning of sexual activity (y)			
< 18	47	**0.0201**	27/20
> 18	27		17/10
Number of sexual partners			
1	15		9/6
> 2	59	**< 0.0001**	35/24
First gynecological examination (y)			
< 18	19		9/10
> 18	55	**< 0.0001**	34/21
First Pap smear (y)			
< 18	13		4/9
> 18	61	**< 0.0001**	36/25
Weight (y)			
< 60	25		15/10
> 60	41	**0.0489**	26/15

*Note:* If the calculated *p*-value is small (*p* < 0.05) of the chi-squared value, then there is a significant difference between the frequencies of the different categories. The clinical data are corresponding only for women with precursor cervical lesions. Bold numbers indicate significant difference.

Abbreviations: y = years, *n* = number.

**Table 2:** Cytology, colposcopy, histology, and human papillomavirus detection in cervical lesions.

Cytology	*n*	Colposcopy	*n*	Histology	*n*	HPV +
NILM	48	Negative	53	Normal	53	8/53
Koilocytosis	8	Condyloma	8	Condyloma	10	8/10
ASCUS	12	CIN1	12	CIN1	7	—
LSIL	32	CIN1	32	CIN1	37	31/44
HSIL	5	CIN2/3	7	CIN2/3	7	5/7
CC	70	CC	70	CC	70	68/70

Abbreviations: ASC-US = atypical squamous cells of undetermined significance, CC = cervical cancer, CIN = cervical intraepithelial neoplasia grades 1, 2 or 3, HSIL = high-grade squamous intraepithelial lesion or CIN2/3, LSIL = low-grade squamous intraepithelial lesion or CIN1, NILM = negative for intraepithelial lesion or malignancy.

**Table 3:** Statistical parameters for NIC2+ and CC devices in cervical lesions.

Statistical parameters	NIC2+ (%)	CC (%)
Sensitivity	94.81 (87.23–98.57)	97.14 (90.06–99.65)
Specificity	51.28 (41.87–60.63)	54.39 (44.79–63.74)
Positive predicted value	56.15 (51.36–60.84)	56.67 (51.60–61.60)
Negative predicted value	93.75 (85.04–97.54)	96.88 (88.67–99.19)
Positive likelihood ratio	1.95 (1.60–2.36)	2.13 (1.74–2.61)
Negative likelihood ratio	0.1 (0.04–0.27)	96.88 (88.67–99.19)
Accuracy	68.56 (61.52–75.02)	70.65 (63.50–77.12)

*Note:* Parenthesis indicates confidence interval at 95%.

## Data Availability

Some more raw data can be found in Mauricio Salcedo doi. 10.5281/zenodo.11002521.
